# Lyssavirus Surveillance in Bats, Bangladesh

**DOI:** 10.3201/eid1203.050333

**Published:** 2006-03

**Authors:** Ivan V. Kuzmin, Michael Niezgoda, Darin S. Carroll, Natalie Keeler, Mohammed Jahangir Hossain, Robert F. Breiman, Thomas G. Ksiazek, Charles E. Rupprecht

**Affiliations:** * Centers for Disease Control and Prevention, Atlanta, Georgia, USA;; † International Centre for Diarrheal Disease and Research, Centre for Health and Population Research, Dhaka, Bangladesh

**Keywords:** lyssavirus, Khujand virus, Aravan virus, rabies, bat, rhabdovirus, Bangladesh, tropical Asia, antibody, dispatch

## Abstract

Lyssavirus surveillance in bats was performed in Bangladesh during 2003 and 2004. No virus isolates were obtained. Three serum samples (all from *Pteropus giganteus*, n = 127) of 288 total serum samples, obtained from bats in 9 different taxa, neutralized lyssaviruses Aravan and Khujand. The infection occurs in bats in Bangladesh, but virus prevalence appears low.

Bats are known reservoirs of viruses in the *Lyssavirus* genus, family *Rhabdoviridae*. In the Americas, bats maintain the circulation of different lineages of rabies virus (RABV, genotype 1) (*1*). Lagos bat virus (genotype 2) and Duvenhage virus (DUVV, genotype 4) were isolated from bats in Africa (*2*). European bat lyssaviruses, types 1 and 2 (EBLV-1 and EBLV-2, genotypes 5 and 6, respectively) circulate in European bat populations (*3*). West Caucasian bat virus, a new putative lyssavirus genotype, was recently isolated from a bat in southern Europe (*4*). Australian bat lyssavirus (ABLV, genotype 7) was isolated from different genera of Australian bats (*5*).

Data on rabies in Asian bats are limited because of a lack of a suitable surveillance system. Only a few investigators reported presumable RABV isolates of bat origin in India and Thailand, but these were not corroborated, nor were reports about bat rabies in Siberia and Uzbekistan (*6*). No confirmed genotype 1 lyssavirus isolates are available from bats outside the Americas, to date. Recently, 3 lyssaviruses (Aravan, Khujand [6], and Irkut [4] viruses) were isolated from bats in different locations of Asia. These representatives were suggested as 3 new putative genotypes of the *Lyssavirus* genus, according to their genetic properties. Moreover, antibodies to lyssaviruses have been demonstrated in serum specimens of bats from the Philippines, Cambodia, and Thailand (*7**–**9*). In this article, we extend information on the geographic distribution of rabies among Asian bats and describe a limited survey in Bangladesh for evidence of lyssavirus activity.

## The Study

**Figure 1:**
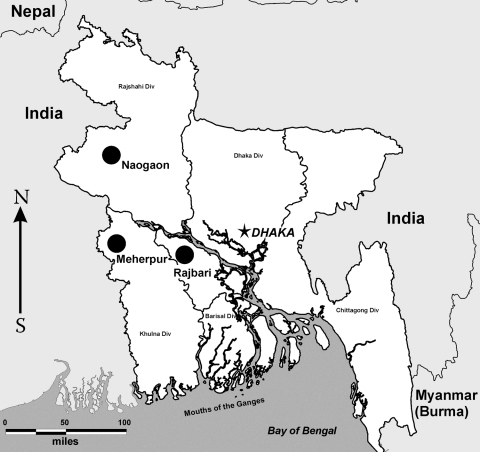
Map of Bangladesh with bat surveillance regions indicated (circles).

This project began as part of a larger study concerned with a suspected Nipah virus outbreak in the region. Active surveillance of bats was performed in 3 districts of Bangladesh: Meherpur and Naogaon during March 2003 and Rajbari during February and March 2004 (Figure 1). The animals were collected randomly from different roosts, trees, and fruit plantations.

Bats were anesthetized by a 0.05- to 0.1-mg intramuscular injection of ketamine hydrochloride. Injured bats and those that had clinical signs and symptoms were euthanized under sedation by exsanguination. Blood was transferred to serum separator tubes and was refrigerated until centrifugation. Serum was decanted into individual screw-topped vials. When possible, bats were identified to species. Brains of all euthanized bats were removed at necropsy and placed into individual sterile containers. Additional organs and oral swabs were also obtained from each bat. All specimens were held in temporary storage at –18°C (after collection) and later placed at –80°C. Carcasses of representative specimens were placed in formalin for archival purposes.

Bat brains collected in 2003 (N = 212, Table) were tested by the direct fluorescent-antibody test (DFAT) (*10*), by using both monoclonal (Centocor Inc., Malvern, PA, USA) and polyclonal (Chemicon International, Temecula, CA, USA) fluorescein isothiocyanate–labeled anti–rabies virus antibodies, and were subsequently processed for isolation by the mouse inoculation test (MIT) (*11*). If death occurred during the MIT, mouse brains were subjected to the DFAT, and a second intracerebral passage was conducted with 5% brain suspensions, using 0.22-μm filters (Millipore Corp., Bedford, MA, USA). Bat brains collected in 2004 (N = 151, Table) were subjected to the DFAT only.

**Table:** Samples of bats captured in Bangladesh and subjected to lyssavirus diagnosis, isolation, and antibody detection*

Species	Brains		Sera	
	DFAT and MIT	DFAT only	1:20†	1:50
*Pteropus giganteus*	37	97	123	4
*Cynopterus sphinx*	6	5	6	1
*Macroglossus sobrinus*	0	1	1	0
*Rousettus leshenaulti*	0	46	11	0
*Megaderma lyra*	47	2	24	23
*Pipistrellus sp.*	85	0	24	37
*Scotophilus heathii*	30	0	15	12
*S*. *kuhlii*	1	0	1	0
*Taphozous saccolaimus*	6	0	2	4

*DFAT, direct fluorescent antibody test; MIT, mouse inoculation test. †Starting dilutions available for rapid fluorescent focus inhibition test.

The presence of virus-neutralizing antibodies was determined by an adaptation of the rapid fluorescent focus inhibition test (RFFIT), as described (*7*). Because of volume limitations and the cytotoxicity of some specimens, 207 serum samples were available for testing at a starting dilution of 1:20, and 81 samples at a starting dilution of 1:50 (Table). Four different lyssaviruses were used in an initial in vitro screening: Aravan, Khujand, Irkut, and ABLV. When a positive result was obtained, testing on the sample was repeated, and comparative assays undertaken for additional lyssaviruses: EBLV-1, EBLV-2, DUVV, and RABV (e.g., routine rabies challenge virus standard, CVS-11). The dose of each virus used for the RFFIT was ≈50 infectious units per 100 μL. The duration of the test was 20 hours for ABLV and CVS-11 and 40 hours for other challenge viruses.

No evidence of lyssavirus antigen was detected in any bat brain by DFAT, and no neurotropic viruses were isolated by mouse inoculation. If a limited number of deaths occurred during the initial MIT (1 or 2 mice of 5 infected), those effects were not reproduced during the subpassage by filtration, which suggests that bacterial contamination of the field samples caused the death of mice during the initial MIT.

**Figure 2:**
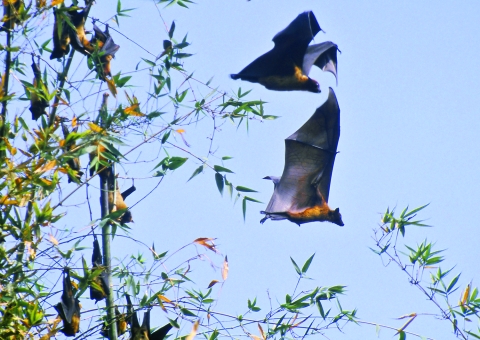
Giant Indian flying foxes (*Pteropus giganteus*). (Photo by I.V. Kuzmin).

One serum sample repeatedly demonstrated neutralizing activity against Khujand virus, at a titer of 54. This sample was obtained from a young female giant Indian flying fox (*Pteropus giganteus*) (Figure 2) captured in the Meherpur District in 2003. Two other serum samples obtained from the same species (1 male and 1 female) in Rajbari District in 2004, neutralized Aravan and Khujand viruses at titers of 14–16. For these latter samples, neutralization was detected at dilutions of 1:20 and 1:25 but not detected at a dilution of 1:50. The titers of all serum specimens were <10 (i.e., no neutralization occurred in the dilution of 1:20) against other lyssaviruses.

## Conclusions

We have presented serologic evidence of lyssavirus infection in bats from Bangladesh. Antigenic cross-reactivity has been reported among Aravan, Khujand, and other members of the *Lyssavirus* genus (*12*). Therefore, detectable antibody may cross-react with other related lyssaviruses, as well as with viruses yet to be discovered.

Based upon this preliminary survey, a low prevalence of lyssavirus infection appears in *P*. *giganteus* in Bangladesh. Thus, it is not surprising that all brain samples collected were negative for detection of lyssavirus antigen. Because different lyssaviruses and bat species are found in Asia, and therefore different virus-host interactions would be expected in the region, extrapolating antibody-positive/virus-positive ratios, as have been estimated from American (*13**,**14*) or European (*15*) bat populations, would be difficult.

Further surveillance for Asian bat lyssaviruses should be conducted. Public health authorities need to be aware of the potential for bats to transmit lyssaviruses, and public education of this potential should be enhanced. Frugivorous bats forage in fruit plantations in many regions of the Old World tropics, including Bangladesh. Indigenous people capture and may consume these animals. Direct contact between humans and bats frequently occurs during these interactions. Absence of current information on human rabies after bat exposure may be a result of inadequate education, incomplete surveillance, and lack of characterization of viruses from rabies cases (*6*).

## References

[1] Smith JS, Orciari LA, Yager PA, Seidel HD, Warner CK. Epidemiologic and historical relationships among 87 rabies virus isolates as determined by limited sequence analysis. J Infect Dis. 1992;166:296–307. 10.1093/infdis/166.2.296 1634801

[2] King A, Crick J. Rabies-related viruses. In: Campbell JB, Charlton LM, editors. Rabies. Boston: Kluwer Academic Publishers; 1988. p. 178–99.

[3] Amengual B, Whitby JE, King A, Serra Cobo J, Bourhy H. Evolution of European bat lyssaviruses. J Gen Virol. 1997;78:2319–28. 9292021 10.1099/0022-1317-78-9-2319

[4] Botvinkin AD, Poleschuk EM, Kuzmin IV, Borisova TI, Gazaryan SV, Yager P, Novel lyssaviruses isolated from bats in Russia. Emerg Infect Dis. 2003;9:1623–5. 14720408 10.3201/eid0912.030374 PMC3034350

[5] Guyatt KJ, Twin J, Davis P, Holmes EC, Smith GA, Smith IL, A molecular epidemiological study of Australian bat lyssavirus. J Gen Virol. 2003;84:485–96. 10.1099/vir.0.18652-0 12560583

[6] Kuzmin IV, Orciari LA, Arai YT, Smith JS, Hanlon CA, Kameoka Y, Bat lyssaviruses (Aravan and Khujand) from Central Asia: phylogenetic relationships according to N, P and G gene sequences. Virus Res. 2003;97:65–79. 10.1016/S0168-1702(03)00217-X 14602198

[7] Arguin PM, Murray-Lillibridge K, Miranda ME, Smith JS, Calaor AB, Serologic evidence of Lyssavirus infections among bats, the Philippines. Emerg Infect Dis. 2002;8:258–62. 10.3201/eid0803.010330 11927022 PMC2732470

[8] Reynes JM, Molia S, Audry L, Hout S, Ngin S, Walston J, Serologic evidence of lyssavirus infection in bats, Cambodia. Emerg Infect Dis. 2004;10:2231–4. 15663870 10.3201/eid1012.040459 PMC3323374

[9] Lumlertdacha B, Boongird K, Sawai Wanghongsa S, Wacharapluesadee S, Chanhome L, Khawplod P, Survey for bat lyssaviruses, Thailand. Emerg Infect Dis. 2005;11:232–6. 15752440 10.3201/eid1102.040691 PMC3320458

[10] Dean DJ, Abelseth MK, Atanasiu P. The fluorescent antibody test. In: Meslin F-X, Kaplan MM, Koprowski H, editors. Laboratory techniques in rabies. Fourth edition. Geneva: World Health Organization; 1996. p. 88–93.

[11] Koprowski H. The mouse inoculation test. In: Meslin F-X, Kaplan MM, Koprowski H, editors. Laboratory techniques in rabies. Fourth edition. Geneva: World Health Organization; 1996. p. 80–6.

[12] Hanlon CA, Kuzmin IV, Blanton J, Manangan J, Murphy S, Rupprecht CE. Efficacy of rabies biologics against new lyssaviruses from Eurasia. Virus Res. 2005;111:44–54. 10.1016/j.virusres.2005.03.009 15896401

[13] Price JL, Everard COR. Rabies virus and antibody in bats in Grenada and Trinidad. J Wildl Dis. 1977;13:131–4. 864845 10.7589/0090-3558-13.2.131

[14] Steece R, Altenbach JS. Prevalence of rabies specific antibodies in the Mexican free-tailed bat (*Tadarida braziliensis mexicana*) at Lava Cave, New Mexico. J Wildl Dis. 1989;25:490–6. 2681843 10.7589/0090-3558-25.4.490

[15] Serra-Cobo J, Amengual B, Abellan C, Bourhy H. European bat lyssavirus infection in Spanish bat populations. Emerg Infect Dis. 2002;8:413–20. 10.3201/eid0804.010263 11971777 PMC2730232

